# Ecological design of high-performance synthetic microbial communities: from theoretical foundations to functional optimization

**DOI:** 10.1093/ismeco/ycaf133

**Published:** 2025-08-21

**Authors:** Zhihan Wang, Shang Wang, Qing He, Xingsheng Yang, Bo Zhao, Haihan Zhang, Ye Deng

**Affiliations:** State Key Laboratory of Regional Environment and Sustainability, Research Center for Eco-Environmental Sciences, Chinese Academy of Sciences, Beijing 100085, China; Shaanxi Provincial Field Scientific Observation and Research Station of Water Quality in Qinling Mountains, Xi’an University of Architecture and Technology, Xi’an, Shaanxi 710055, China; State Key Laboratory of Regional Environment and Sustainability, Research Center for Eco-Environmental Sciences, Chinese Academy of Sciences, Beijing 100085, China; State Key Laboratory of Regional Environment and Sustainability, Research Center for Eco-Environmental Sciences, Chinese Academy of Sciences, Beijing 100085, China; State Key Laboratory of Regional Environment and Sustainability, Research Center for Eco-Environmental Sciences, Chinese Academy of Sciences, Beijing 100085, China; College of Resources and Environment, University of Chinese Academy of Sciences, Beijing 100049, China; State Key Laboratory of Regional Environment and Sustainability, Research Center for Eco-Environmental Sciences, Chinese Academy of Sciences, Beijing 100085, China; College of Resources and Environment, University of Chinese Academy of Sciences, Beijing 100049, China; Shaanxi Provincial Field Scientific Observation and Research Station of Water Quality in Qinling Mountains, Xi’an University of Architecture and Technology, Xi’an, Shaanxi 710055, China; State Key Laboratory of Regional Environment and Sustainability, Research Center for Eco-Environmental Sciences, Chinese Academy of Sciences, Beijing 100085, China; College of Resources and Environment, University of Chinese Academy of Sciences, Beijing 100049, China

**Keywords:** community assembly, keystone species, metabolic interaction, machine learning, functional optimization

## Abstract

The complexity of natural microbial communities poses significant challenges for predictive manipulation, driving the emergence of Synthetic Microbial Communities (SynComs) as tractable models for functional optimization in environmental, agricultural, and biomedical applications. While SynComs provide enhanced controllability, their rational design faces persistent challenges in achieving both functional precision and ecological stability. Here, we present a theoretical and methodological framework for engineering SynComs through the strategic integration of ecological principles, evolutionary theory, and computational innovation. By (i) ecological interaction engineering for dynamic equilibrium of cooperative and competitive relationships, (ii) hierarchical species orchestration ensuring structural integrity through keystone species governance, helper-mediated adaptation, and rare taxa preservation, (iii) evolution-guided artificial selection overcoming functional-stability trade-offs, and (iv) modular metabolic stratification for efficient resource partitioning, we demonstrate how SynComs can be programmed for predictable functionality. We further identify critical frontiers for SynCom construction and application, including: mechanistic decoding of microbial interaction networks, high-throughput culturomics for strain discovery, artificial intelligence-enabled exploitation of microbial dark matter, automated platform-assisted consortium assembly, predictive modelling of long-term community dynamics, and the development of standardized frameworks and shared databases. The theory-technology integrated paradigm establishes SynComs as programmable ecotechnologies capable of addressing global sustainability challenges through engineered ecological resilience. This synthesis provides both a conceptual roadmap and a practical toolkit for transitioning from empirical community construction to predictive ecosystem engineering.

## Introduction

Research on synthetic microbial communities (SynComs) has emerged from advances in microbial ecology, high-throughput isolation techniques, and the growing demand for microbial resource utilization [1]. Breakthroughs in genetic engineering and synthetic biology enable the rational design of artificial microbial consortia with tailored functions, demonstrating significant potential in environmental remediation (e.g. pollutant degradation) [2], biomedicine (e.g. microbiome-based therapies) [3], agriculture (e.g. plant growth-promoting consortia) [4], and industrial biotechnology (e.g. efficient biomanufacturing) [5].

**Figure 1:**
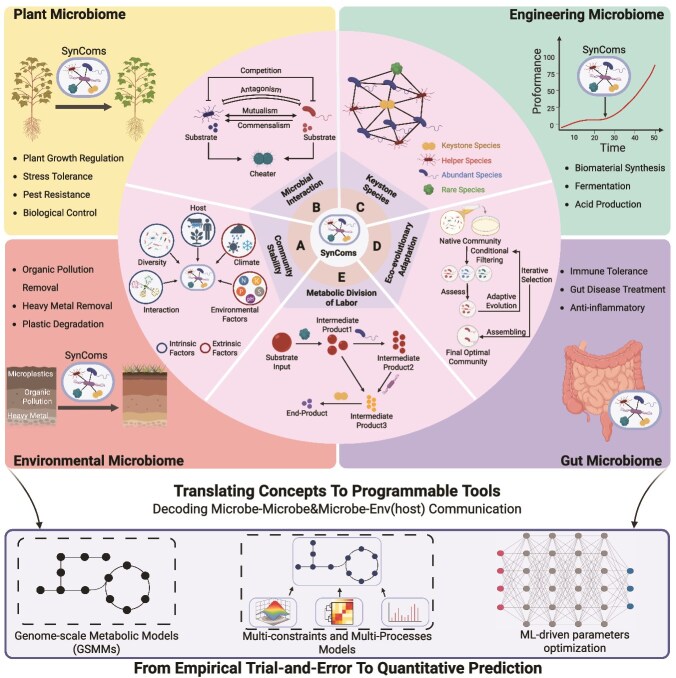
Ecological theories guiding the rational design of SynComs. Theoretical frameworks in community ecology, including microbial stability theory (A), microbial interaction theory (B), keystone species theory (C), eco-evolutionary adaptation (D) and metabolic division of labor (E), converging with advanced modeling facilitate the transition from empirical assembly to predictive design. Created in BioRender.

Despite the growing demand and demonstrated potential of SynComs, their functional efficacy remains inconsistent in target environments. Agricultural applications, for instance, show variable performance depending on temporal dynamics, climatic variations, and edaphic factors [6]. This inconsistency highlights the crucial need for a mechanistic understanding and science-based design principles to improve a SynCom’s reliability and effectiveness in real-world settings. Emerging ecological principles are providing a theoretical foundation for rational SynCom design and functional optimization (Fig. 1). Key insights include: community stability theory, which identified broad principles governing community dynamics in engineered microbial systems; microbial interaction networks including mutualism, antagonism, and commensalism, which shape consortium robustness and functional output; keystone species theory, which enhances plant growth promotion or bioproduction efficiency by optimizing synergistic colonization and ecological robustness [7]; evolutionary theory, which guides strain selection by predicting long-term adaptation beyond short-term abiotic constraints (e.g. pH, nutrient gradients) [8, 9]; metabolic division of labor, which improves substrate conversion efficiency through coordinated pathway allocation [10, 11]. These principles are now being operationalized through an integrated computational and experimental framework (Fig. 1).

The convergence of advanced modeling and empirical validation has enabled a paradigm shift from trial-and-error to rational design. Genome-scale metabolic networks (GSMMs), enhanced with multidimensional constraints (kinetic, thermodynamic, multi-omics), translate ecological theories into predictive models. Machine learning further accelerates this process by optimizing parameters and interaction predictions [12]. This computational power synergizes with experimental validation through multi-omics analysis and automated high-throughput screening, creating a robust and accelerated design-build-test-learn cycle (DBTL) [13]. DBTL is an iterative engineering framework comprising four stages: Design (Computational prediction of interaction networks), Build (Assembly of defined microbial consortia), Test (Functional validation under target conditions), and Learn (Data-driven model refinement). Critically, the effectiveness of this framework is amplified by strategically combining two complementary approaches: (i) top-down refinement of natural communities preserves evolved ecological relationships, while (ii) bottom-up trait-targeted assembly enables precise functional programming [14]. Together, these advances are establishing SynCom engineering as a predictable discipline with significantly higher success rates.

## Ecological theories guiding SynComs’ design

### Microbial community stability and SynComs stability optimization

Microbial community stability encompasses multiple dimensions with distinct ecological implications. This includes resistance (the ability to withstand disturbance without significant functional or compositional shifts), resilience (the capacity of the microbiome to recover from perturbation), and their combination—robustness (the ability of a microbial community to maintain its structural organization and functional performance in the face of disturbances) [15]. Microbial community stability following disturbance is governed by an interplay between intrinsic and extrinsic factors (Fig. 1A). Intrinsic factors include microbial diversity, inter-species interactions, and species-host feedbacks, whereas extrinsic factors include abiotic conditions and climate regimes [16]. The relationship between diversity and stability is, however, context-dependent [17]. Although high diversity frequently enhances stability, exemplified by gut microbiomes with increased pathogen resistance [18], elevated richness can intensify resource competition under nutrient limitation, thereby reducing both stability and functionality [16]. Importantly, several ecosystems retain functionality despite biodiversity loss, relying on compensatory mechanisms like metabolic redundancy [19] or species plasticity [20]. Furthermore, microbial interactions not only shape immediate community dynamics but also drive long-term evolutionary trajectories that promote stable coexistence [21]. At the plant-microbe interface, root exudates and rhizodeposits have been shown to stabilize rhizosphere bacterial communities [22]. Extrinsic drivers exert equally decisive control. For instance, long-term experimental warming in grassland ecosystems significantly increased microbial network stability and ecosystem functional potentials [23], whereas soil organic carbon content modulate both microbial community stability and species interactions [24]. Collectively, these findings underscore that microbial community stability emerges from a dynamic interplay between endogenous biotic processes and exogenous environmental drivers.

In SynComs, determining whether a specific combination of strains can stably co-exist in an artificial co-culture system over time is a priority focus [25], and some research has also argued that the stability assessment should consider functional retention rather than species persistence alone [25]. However, critical gaps persist in SynCom stability assessment—only 40% of studies (35/86) evaluate stability, neglecting longitudinal monitoring of community performance in field conditions [25]. Stability emerges from species composition, functional diversity, and interspecies interactions [26], which SynComs leverage through cross-trophic interactions or native species integration [27–29]. SynComs’ design efforts must balance diversity-functionality trade-offs: high-diversity SynComs improve ecological performance but hinder scalability, while over-simplified consortia risk losing keystone species and stability [30–33]. Therefore, optimal SynCom stability requires: (i) spatially structured community allowing division of labor and communication, (ii) balanced diversity via omics-guided keystone species selection, and (iii) dynamic adaptation to disturbances. Notably, computational approaches are revolutionizing SynCom stability optimization, providing practical constraints on SynCom preparation and application through multi-scale modeling frameworks [34–36].

### Microbial interaction theory

Microorganisms establish complex interaction networks through niche partitioning and resource utilization, defining their ecological roles and community dynamics [37, 38]. However, the practical success of SynComs relies critically on how well their constituent members interact—specifically, the stability, scalability, and controllability of these interactions, all of which demand thorough characterization and optimization [39].

Positive interactions (Fig. 1B), including mutualism and commensalism frequently emerge from metabolic specialization, where cross-feeding of metabolic byproducts enhances overall efficiency and resilience [40]. Notably, commensalism predominates under extreme conditions [41], suggesting its critical role in environmental adaptation. Engineered SynComs leveraging such positive interactions demonstrate superior performance. A cross-feeding yeast consortium increased 3-hydroxypropionic acid production through evolved mutualism [42]. Modular metabolic SynComs achieved efficient chitosan degradation through coordinated enzymatic pathways [43]. Agricultural SynComs composed of *Pseudomonas* Leaf15, *Rhizobium* Leaf68, and *Acidovorax* Leaf76 suppressed pathogens through metabolite exchange [44]. Endophytic *Bacillus* SynComs enhanced plant growth via phytohormone production and nutrient provision [45].

Negative interactions in SynComs primarily manifest through competition and antagonism (or amensalism) (Fig. 1B), mediated by both resource competition (e.g. nutrients, space) [46] and chemical warfare [47]. Species compete for limited nutrients and space, leading to dynamic changes in species dominance, as demonstrated by the mutualism-to-competition transition in a *Chlorella vulgaris-Saccharomyces cerevisiae* consortium under elevated NH₄^+^ conditions [48]. Interestingly, even in cooperative systems, competition can emerge upon community expansion. For instance, cross-feeding in a three-member SynCom maximized the yield of 4-ethylclove acid, but the introduction of additional strains triggered competition and reduced efficiency [11]. Antagonistic interactions involve active suppression through antimicrobial compounds, including antibiotics (e.g. polyketides), organic acids (e.g. lactate), bacteriocins (e.g. nisin), and biosurfactants (e.g. rhamnolipids) [47]. A recent study revealed that competitive outcomes are strongly predicted by phylogeny and biosynthetic gene clusters (BGCs) overlap [49], surpassing the explanatory power of simple inhibition assays. Environmental context critically modulates these interactions: under high resource conditions, antibiotics selectively target ecologically similar but genetically distinct competitors, whereas nutrient limitation promotes antibiotic suppression in favor of alternative survival strategies [49]. Notably, phage predation intensifies competition through “kill-the-winner” dynamics by preferentially lysing dominant taxa [50]. Strategic manipulation of competition can enhance community stability, as evidenced by the stabilizing effect of introducing a third competitor species [44].

Beyond these direct positive and negative interactions, microbial interactions also face another critical challenge—cheating behavior. Cheating behavior threatens microbial cooperation by exploiting shared resources without contributing (Fig. 1B), which can lead to a collapse of mutualistic partnerships [51, 52]. Antibiotic pressure further exacerbates cheater dominance [53]. Emerging approaches mitigate cheating through ecological engineering. For instance, iron distribution models demonstrate how differential resource utilization enables cheater–producer coexistence [54]. The deliberate incorporation of spatial organization into microbial community design represents a powerful strategy for enhancing cooperation while suppressing cheating behavior [55, 56], likely due to confined microenvironments altering quorum sensing (QS) dynamics [57] and public goods distribution [58]. Prolonged cheater-producer interactions drive adaptive counterstrategies (e.g. evolved resource hoarding) [59]. Paradoxically, cheaters may enhance community diversity and adaptability in specific contexts [60, 61], as observed in pollutant-degrading SynComs where nonfunctional “hitchhikers”(i.e. cheaters) coexisted with degraders [62].

These interaction paradigms provide critical design guidelines for SynComs: (i) prioritizing metabolically interdependent strains to stabilize mutualism/commensalism; (ii) minimizing parasitic/antagonistic pairs through genomic screening (e.g. phage resistance genes, antibiotic BGC overlap); (iii) engineering spatial or nutrient constraints to suppress cheating. This framework underscores the need to balance interaction types for robust SynCom performance, bridging ecological theory with biotechnological applications.

### Keystone species and functional modularity

Keystone species are crucial for maintaining the structure and function of the community, impacting microbiome functionality independently of their abundance. Jiang *et al.* summarized a series of different methods for identifying keystone species in natural microbial communities [63], among which ecological network analysis is the most popular approach. The selection of strains for SynComs is guided by their ecological roles and functional contributions. Thus, keystone species identification in SynComs should be additionally combined with experimental (species removal) or computational (e.g. GSMM) validation [8, 41]. Compared to the time-consuming experimental validation, GSMM construction was more suitable for screening species with metabolic cooperations from large data and complex communities [8].

Keystone species maintain community stability by mediating interactions with helper species or rare species (Fig. 1C), as revealed by the molecular network analyses [64, 65]. The functional synergy between keystone and helper species amplifies community performance beyond individual capabilities [66]. For example, Xun *et al.* constructed a *Bacillus*-centric SynCom, where *Bacillus* synthesizes the antimicrobial lipopeptide fengycin, while helper species enhanced their efficacy by stimulating growth and upregulating antimicrobial gene expression [67]. Parallel findings by Sun *et al.* showed *Bacillus*-mediated coordination of selenium nanoparticle synthesis coupled with beneficial microbiota recruitment [68]. These findings demonstrate that keystone species function as ecological orchestrators, coordinating helper species into synergistic functional modules via sophisticated interspecies communication.

Generally, numerically abundant species sustain essential metabolic fluxes and thus ecosystem functions [69]. A recent study showed that not all keystone species identified through abundance shifts are functionally essential [63]. Ruan *et al.* [8] showed that ecological filtering (e.g. herbicide-driven selection) enriched 18 candidate keystone strains, but only four, including *Bacillus* sp. P56, *Lysinibacillus* sp. LM5, *Acinetobacter* sp. AC6, and *Bradyrhizobium* sp. BR1 significantly enhanced consortium growth and degradation efficiency. The remaining taxa, despite their increased abundance under selective pressure, either contributed minimally or competed for resources [8], highlighting that abundance shifts alone are insufficient proxies for functional importance.

Within abundance gradients, rare taxa may additionally provide disproportionate contributions to community resilience [70]. Thus abundant and rare taxa may exhibit functional partitioning through: (i) the “Matthew effect”, where rare species counterbalance dominant members to maintain metabolic diversity [71]; (ii) functional complementarity, as demonstrated in drought-tolerant agave systems where inoculation of abundant-species-derived SynComs into agave rhizosphere soils elevated rare species’ abundance, subsequently enhancing drought tolerance via abundant-rare taxa interactions [72]; and (iii) strategic division of labor, as seen in plant protection systems where abundant species directly suppress pathogens while rare taxa activate host immune responses [73].

The rational construction of functional SynComs requires a systematic understanding of the complementary functional roles within microbial ecosystems. Keystone species provide essential functional foundations, helper species amplify these functions [67], and rare species offer critical insurance against environmental fluctuations [73]. This functional stratification informs three core design principles essential for robust SynCom engineering. First, strain selection must prioritize metabolic network connectivity over simple taxonomic composition. Second, dynamic equilibrium among functional groups must be maintained through balanced population ratios. Third, strategic niche space allocation preserves the ecological flexibility required for environmental adaptation. This integrated approach underscores the necessity of integrating functional validation with abundance-based screening in SynCom design [74].

### Eco-evolutionary adaptation in SynCom engineering

Community assembly represents a dynamic ecological process whereby microbial species coalesce into functional consortia through both ecological and evolutionary mechanisms [75]. Artificial selection has emerged as a powerful directed strategy for enhancing microbial community functionality through iterative selection [76] (Fig. 1D). However, its application in microbiome engineering remains constrained by variable success rates and limited functional improvements, primarily due to the lack of efficient selection strategies tailored for complex microbial systems. For example, bacterial mutualism in the plant rhizosphere can rapidly evolve through enhanced metabolic adaptation and reciprocal feedback with hosts [77], highlighting that rational SynCom design must account for strains’ evolutionary potential rather than relying solely on their initial functions.

Recent advances integrating ecological and evolutionary theory have opened new avenues for optimizing SynCom composition. The pioneering work by Arias-Sánchez *et al.* established a genetic algorithm-inspired framework for directed community evolution [78]. After 18 rounds of selection and recombination, stochastically assembled four-member communities evolved pollutant degradation efficiencies surpassing initial optimal communities [9]. Using this evolutionary concept, Chang *et al.* introduced computational modeling to simulate community dynamics under iterative selection and stochastic perturbations, creating a powerful platform for top-down engineering of complex, diverse consortia [36]. Most recently, Ruan *et al.* bridged the gap between top-down and bottom-up strategies through an integrated pipeline combining machine learning for keystone species identification with SuperCC metabolic modeling, enabling precision engineering of functionally enhanced communities [8].

At the technological frontier, directed selection is being revolutionized by advanced genetic engineering tools. Precise genome editing and synthetic pathway integration now enable the creation of specialized microbial chassis with tailored functions [79, 80]. While these methods demonstrate considerable promise in synthetic biology applications, persistent limitations remain in functional enhancement efficacy and operational complexity [25]. Artificial directed selection bridges theory and practice through three principal technological paradigms: top-down ecology-informed directed evolution for emergent functionality, bottom-up precision genome editing for targeted trait engineering, and hybrid computational model-driven optimization integrating machine learning with metabolic modeling or genomic modeling. This multiscale approach fundamentally advances microbial community regulation by uniquely addressing the “function-stability trade-off” in complex SynComs, providing interoperable solutions that bridge evolutionary principles to molecular design, and enabling the interdisciplinary convergence of ecological theory, artificial intelligence, and synthetic biology. Future breakthroughs will require a deeper integration of these paradigms to overcome functional enhancement bottlenecks, particularly in bridging fundamental mechanisms with scalable engineering applications.

## Metabolism-focused optimization and computational tools for SynComs

### Strategies for metabolic division of labor

The metabolic division of labor represents a fundamental ecological strategy in microbial communities, where distinct populations specialize in specific biochemical tasks to collectively achieve complex metabolic functions (Fig. 1E) [81]. This emergent property arises from evolutionary processes, including speciation, genetic diversification, and ecological interactions (competition, cooperation, and cheating) [82], with human intervention further modulating these dynamics [83]. Metabolic division of labor manifests through four principal forms (Table 1): Division of Labor (DOL) [84], Static Division of Labor (SDOL) [10], Metabolic Division of Labor (MDOL) [85], and Dynamic Division of Labor (DDOL) [86].

**Table 1:** Core characteristics and conceptual evolution of metabolic division of labor in microbial communities.

**Division type**	**Core characteristics**	**Theoretical contribution**	**Dependent technologies/methods**
DOL	Fundamental concept describing functional specialization among strains	Establishes the theoretical framework of “functional differentiation”	Traditional culturing, 16S rRNA sequencing, metabolite detection
SDOL	Genetically or environmentally fixed division (e.g. obligate symbiosis)	Reveals stable division patterns from long-term evolution/environmental selection	Metagenomics, comparative genomics
MDOL	Step-wise partitioning of linear metabolic pathways (e.g. A → B → C by different strains)	Quantifies metabolic flux distribution (e.g. m/n ratio) and predicts stability	Genome-scale metabolic models (GSMM), flux balance analysis
DDOL	Dynamic role switching under environmental fluctuations (e.g. metabolic reprogramming under stress)	Introduces temporal dimension to explain community adaptability	Single-cell tracking (microfluidics), real-time metabolomics

**Figure 2:**
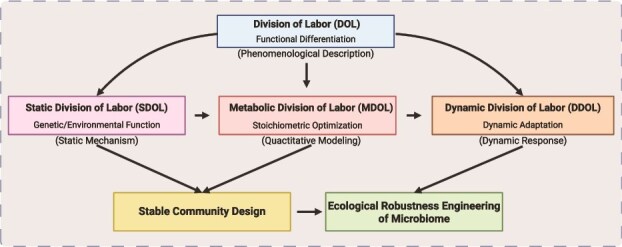
The conceptual evolution of metabolic division of labor and its potential application in SynCom development. Created in BioRender.

The conceptual evolution of metabolic division of labor in microbial communities has progressed from foundational phenotypic observations to sophisticated dynamic frameworks (Fig. 2; Table 1), reflecting deeper mechanistic understanding and technological advancements. Initially, DOL established the basic paradigm of functional specialization among microbial populations, while SDOL revealed how genetic fixation and environmental constraints maintain stable cooperative relationships. The development of MDOL introduced quantitative precision through genome-scale metabolic modeling and flux balance analysis, enabling the prediction of optimal metabolic flux distributions in linear pathways. Most recently, DDOL has expanded this framework by incorporating temporal adaptation to environmental fluctuations through single-cell resolution techniques like microfluidics and real-time metabolomics. This progression—from descriptive (DOL) to mechanistic (SDOL), quantitative (MDOL), and ultimately adaptive (DDOL) perspectives—has been paralleled by technological advances from traditional culturing to multi-omics integration. The complementary nature of these frameworks now enables both the design of stable consortia (through SDOL/MDOL principles) and environmentally responsive systems (via DDOL approaches) (Fig. 2), providing a comprehensive toolbox for engineering microbial communities that balance functional efficiency with ecological robustness in fluctuating environments.

Engineering applications demonstrate that metabolic division of labor can enhance SynCom performance substantially [87]. For example, Shin *et al.* engineered a DOL-based SynCom for sugar fermentation, developing an ordinary differential equation model that integrates substrate utilization kinetics, population growth dynamics and metabolite flux regulation [88]. Experimental validation demonstrated superior performance of SynCom to monocultures, with ~40% faster substrate conversion and ~ 25% higher ethanol yield [87]. Du *et al.* achieved a 3.2-fold increase in 3-(methylthio)-1-propanol production via SDOL engineering, significantly improving liquor flavor profiles [89]. Lin’s MDOL-designed consortium enabled 78% lignocellulose conversion efficiency [14].

Current research has established actionable engineering principles, including pathway modularization [90], optimized metabolite transfer [91], and quorum-regulated role switching [92]. These principles transform MDOL from a theoretical concept to a practical engineering tool. Emerging multi-omics analytics and computational modeling now enable precise metabolic programming of microbial consortia, paving the way for advanced biomanufacturing and environmental applications.

### Model-driven community metabolic optimization

Metabolic interactions serve as a fundamental strategy for functional optimization in SynComs. Metabolic interactions (e.g. cross-feeding, syntrophy) and division of labor (e.g. pathway specialization) require precise coordination among microbial populations to optimize resource use and fitness [40, 93, 94]. Such dynamic metabolic coupling cannot rely solely on passive diffusion of metabolites; instead, it demands active communication via QS [95], which links environmental sensing to population-wide metabolic decisions. From an evolutionary perspective, metabolic dependencies may arise from loss-of-function mutations or genome streamlining (the “Black Queen Hypothesis”) [96], providing a theoretical foundation for engineering interdependent microbial consortia. Modern synthetic biology tools now enable the precise construction of auxotrophic strains [97] and the design of programmable metabolic circuits [98], thereby establishing controlled metabolic interactions within SynComs. It is demonstrated that metabolic interaction networks not only enhance functional output but also improve community stability.

Breakthroughs in computational modeling have revolutionized the design of metabolic interactions in microbial communities [99]. The integration of GSMMs with machine learning algorithms allows for the predictive optimization of community metabolic networks [100, 101]. Thommes *et al.* [102] developed a constraint-based reconstruction and analysis approach for *E. coli* consortia, demonstrating that optimized MDOL through strategic pathway partitioning could boost theoretical yields by 35%–40% while minimizing metabolic interference. Their simulations particularly highlighted how the spatial arrangement of auxotrophic strains reduces metabolite leakage. Community-scale metabolic modeling based on species genomic data has emerged as an active research frontier [103]. Oftadeh *et al.* developed yeast Energy and Thermodynamics Flux, the first genome-scale metabolic model for *S. cerevisiae* that integrates metabolic networks with gene expression constraints and reaction thermodynamics. This Metabolic and Expression Model (ME-models) enables the quantitative analysis of metabolic fluxes, enzyme expression, and energy costs at single-cell level resolution and can be extended to predict community-level behaviors like growth synchronization and phenotypic heterogeneity through multi-species integration [104]. Espinel-Ríos *et al.* [105] pioneered an integrated machine learning platform that combines genome-scale metabolic modeling, Bayesian neural networks, and genetic algorithms, achieving over 90% prediction accuracy for metabolic networks and a more than 6-fold improvement in design efficiency for SynComs. Peng *et al.* [41] developed an innovative GSMM-RMT framework that analyzes metagenome-assembled genomes to identify metabolic dependencies and potential nutrient exchanges among co-occurring species. Ruan *et al.* [8] developed an integrated multi-species model combining individual GSMMs, enabling predictive analysis of community performance under diverse nutritional conditions. The optimized model utilizes flux balance and variability analyses to inform experimental design, enabling the selection of optimal species combinations and cultivation parameters to achieve targeted biotechnological outcomes [8]. These computational approaches improve construction efficiency and offer new insights into complex microbial interactions.

The field is advancing along three key directions: (i) developing in situ metabolic tracking technologies for real-time monitoring of interactions, (ii) establishing automated platforms to accelerate consortium assembly, and (iii) extending current methodologies to nonmodel microbial systems. The true innovation of metabolic interaction design lies in its interdisciplinary integration of evolutionary theory, synthetic biology, and computational modeling.

## Technological challenges and innovative solutions for high-performance SynCom

**Figure 3:**
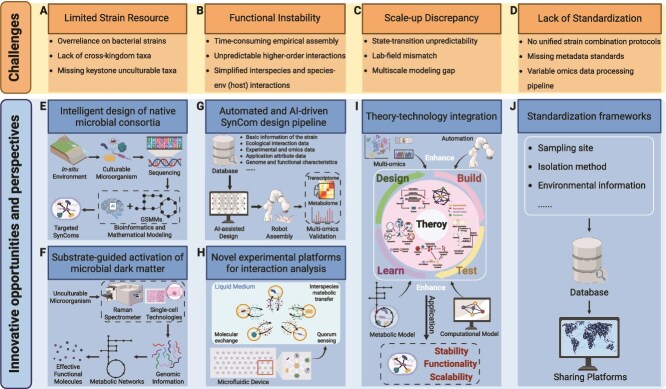
Challenges (A–D), opportunities and future perspectives (E–J) in SynCom construction and application. Created in BioRender.

Ecological theory provides a powerful framework for accelerating SynCom development. This is exemplified by a herbicide-degrading consortium [8], where three key ecological principles were systematically applied to transform complex soil microbiomes into functional consortia: (i) environmental filtering directed the top-down selection of functional communities; (ii) keystone species theory enabled bottom-up design; (iii) metabolic division of labor optimized species interaction to achieve high performance [8]. The study developed the SuperCC modeling platform to operationalize these principles by predicting optimal strain combinations and narrowing down 290 initial isolates to four functional keystones. It also simulated metabolic cross-feeding networks, which were subsequently validated through multi-omics analyses [8]. The case exemplifies how ecological theory, when integrated with computational modeling and targeted functional validation, can overcome traditional trial-and-error approaches in microbiome engineering. Despite these advances, the field continues to face significant limitations, such as limited strain resources (overreliance on culturable strains neglects ecologically critical unculturable taxa) (Fig. 3A), functional instability (empirical assembly methods poorly predict long-term interaction dynamics) (Fig. 3B), scale-up discrepancy (laboratory-optimized consortia frequently fail in field conditions) (Fig. 3C), and lack of standardization (inconsistent protocols hinder reproducibility and cross-study comparisons) (Fig. 3D). Recent advances integrating ecological principles with computational and experimental innovations are needed to address these challenges. In this section, we highlight key considerations and future perspectives for SynCom development and applications (Fig. 3).

### Harnessing indigenous and unculturable microbes

Current SynComs predominantly rely on a limited range of bacterial strains, exhibiting poor environmental adaptability and imprecise functional characterization. The conventional screening approaches often fail to accurately identify key functional taxa due to (i) over-reliance on culturable microorganisms, (ii) lack of cross-kingdom taxa, and (iii) missing keystone unculturable taxa (Fig. 3A). This strain resource limitation severely compromises SynComs’ capacity to establish stable ecological relationships with indigenous microbiomes, leading to frequent functional collapse in field applications.

The strategic use of indigenous microorganisms presents a transformative opportunity for enhancing SynCom performance through intelligent microbial matchmaking (Fig. 3E). This paradigm integrates: (i) artificial intelligence (AI)-assisted selection of phylogenetically compatible strains from native microbiomes using network analysis tools [12]; (ii) rational design of interspecies or cross-kingdom consortia (e.g. fungal-bacterial partnerships [106]) based on complementary metabolic fingerprints; and (iii) dynamic optimization of strain ratios through computational modeling to predict interaction outcomes (Fig. 3E). Such rationally designed consortia could demonstrate superior environmental persistence and functional robustness compared to conventional assemblages of a single group of organisms.

Emerging integrated approaches of single-cell technologies and multi-omics analyses can enable functional activation of uncultivable microorganisms (Fig. 3F). This innovative pipeline involves: (i) identification of metabolically active cells via Raman-activated sorting coupled with single-cell sequencing [107]; (ii) reconstruction of genome-scale metabolic models at single-cell resolution or metabolic networks at community-level using bioinformatics pipelines such as iNAP [41, 108] or SuperCC [8]; (iii) environmental metabolomics to identify specific effector molecules (e.g. rare glycosides or modified amino acids) [109]; and (iv) development of stimuli-responsive delivery systems (e.g. pH-triggered nanoparticles) for targeted substrate release [110]. The key advancement lies in establishing a new paradigm to overcome microbial uncultivability limitations. However, it is important to note that microbial behavior often diverges between mono-culture and multi-culture contexts, necessitating careful consideration when extrapolating single-cell predictions to community-level interactions.

### Accelerating SynCom development: advanced automation technologies and high-throughput experimental validation

Traditional manual assembly of synthetic communities is labor-intensive and slow, often requiring months of iterative culturing, cross-streaking and validation for each strain pair (Fig. 3B). The integration of advanced automation technologies is revolutionizing and accelerating the entire SynCom development pipeline from design to validation. This comprehensive approach combines (i) AI-assisted in silico design of functionally optimized consortia, (ii) robotic assembly systems for high-precision strain combination, (iii) microfluidic cultivation platforms enabling parallelized testing of hundreds of community variants, and (iv) automated metabolomic/transcriptomic analysis for rapid performance evaluation (Fig. 3G). Such integrated workflows can compress traditional development cycles from months to weeks while improving functional predictability [111]. This data-generating pipeline feeds directly into experimental validation systems, creating a continuous improvement cycle.

SynComs frequently exhibit functional instability emerging from unanticipated interspecies dynamics (e.g. complex higher-order and cross-trophic interactions) under environmental fluctuations (Fig. 3B). Innovative tools like the μCI co-cultivation device [111] enable the systematic analysis of complex microbial interactions (Fig. 3H). These platforms integrate microfluidics, 3D printing [112], and microencapsulation [113] to create spatially structured environments that maintain physical separation while allowing controlled chemical communication, enabling precise community composition adjustment, and improving colonization success rates. This combined approach allows researchers to study and optimize SynCom behavior under realistic yet controlled conditions, facilitating the construction of more stable and functional microbial communities. The technology bridges the gap between laboratory studies and natural ecosystem applications.

### Bridging design and implementation in synthetic microbial communities

The transition of SynComs from laboratory designs to real-world applications faces fundamental challenges stemming from microbial ecosystem complexity (Fig. 3C). A core limitation lies in predicting and controlling critical ecological transitions, such as shifts between stable and oscillatory states, which frequently decouple laboratory-optimized parameters (e.g. species richness, interaction strengths) from actual field performance. Environmental variability further exacerbates this mismatch, rendering many *in vitro*-calibrated consortia functionally ineffective *in situ*. To bridge this gap, next-generation computational frameworks are advancing multi-scale modeling approaches that integrate theoretical predictions with practical implementation constraints, enabling more robust translation of SynCom designs (Fig. 3I).

At the metabolic scale, quantitative modeling provides fundamental insights into community assembly rules [34]. The MDOL framework established that stable metabolic division of labor requires: (i) for linear pathways, initial-step specialists must maintain a growth advantage (“m”) over the private benefit of final-step specialists (“n”); (ii) the stable abundance ratio of final-step specialists equals “n/m” [34]. Validated in naphthalene-degrading systems with <10% prediction error for diverse pathways, this approach bridges empirical observation and engineered design through fundamental kinetic parameters (m, n) that control community stability. Moving to the mechanistic level, the Microbial Consumer Resource Model provides a powerful lens for examining community dynamics. By precisely quantifying species-resource interactions and cross-feeding networks through consumer-resource equations, systematically testing disturbances responses (dilution events, species invasion, and member removal or addition), and predictively modeling hypothetical functional outputs (biomass/metabolite production), this approach identifies critical stability thresholds that guide practical implementation parameters [36]. These parameters enable dynamic stability windows for SynCom operation under disturbances. At the macroscopic scale, coarse-grained theories [35] identify species richness and average interaction strength as key control variables governing phase transitions between distinct ecological states (stable coexistence, partial extinction, and oscillatory states), mirroring thermodynamic principles where a few critical variables (e.g. temperature/pressure) determine system behavior. This coarse-grained model enables strategic precalibration of interaction strengths during design phases and real-time monitoring of species richness and interaction intensity during scale-up. The integration of these multi-scale modeling approaches with high-throughput experimental validation (e.g. culturomics, microfluidics, and single-cell sorting) and an automated pipeline could advance SynCom development towards truly predictive design (Fig. 3I), where stability, functionality, and scalability can be engineered with increasing precision and reliability.

### Demand for standardization frameworks

The field suffers from a notable absence of standardized protocols, with significant variations in community construction methods, data collection procedures, and analytical approaches (Fig. 3D). This lack of standardization substantially impacts the reproducibility and comparability of research findings. The establishment of comprehensive standardization protocols requires systematic development across multiple dimensions [4]. This standardization framework must encompass rigorous design specifications for strain combinations, expanded metadata standards (such as MIxS extensions for inoculation concentrations and carrier types), and strain passports documenting collection GPS coordinates, isolation conditions, and patent status (Fig. 3J). Parallel to these protocols, the construction of modular synthetic ecosystem platforms enables rigorous validation, featuring interchangeable microbial, soil and climate components, as well as high-throughput monitoring systems. This standardization framework will enable critical advancements in SynCom applications by: enhancing experimental reproducibility across research groups, facilitating comparative analysis of community performance, supporting regulatory approval processes and enabling technology transfer and commercialization. Such coordinated action will ensure reproducibility and scalability for real-world applications.

## Conclusions

Recent investigations into SynComs are systematically elucidating the intricate dynamics of microbial interactions while significantly broadening their potential biotechnological applications. Future studies should further investigate internal metabolic relationships governing community succession, high-throughput technologies for rapid strain discovery, automated platforms for efficient community construction, and advanced modeling approaches for dynamic prediction. Key innovations will emerge from integrating ecological theory with synthetic biology tools, enabling precise design of SynComs for environmental and health applications. The development of standardized frameworks and shared databases will be crucial for advancing the field toward predictive community engineering.

## Data Availability

Data sharing not applicable to this article as no datasets were generated or analyzed during the current study.
